# Complete Genome of the Attenuated Sparfloxacin-Resistant *Streptococcus agalactiae* Strain 138spar

**DOI:** 10.1128/genomeA.00431-14

**Published:** 2014-05-15

**Authors:** Julia W. Pridgeon, Dunhua Zhang, Lee Zhang

**Affiliations:** a Aquatic Animal Health Research Unit, USDA, ARS, Auburn, Alabama, USA; b Genomics and Sequencing Laboratory, Department of Entomology and Plant Pathology, Auburn University, Auburn, Alabama, USA

## Abstract

Through the selection of resistance to sparfloxacin, an attenuated *Streptococcus agalactiae* strain, 138spar, was obtained from its virulent parent strain, *S. agalactiae* 138P. The full genome of *S. agalactiae* 138spar is 1,838,126 bp. This genome will allow comparative genomics to identify genes associated with virulence, antibiotic resistance, or other characteristics.

## GENOME ANNOUNCEMENT

The Gram-positive bacterium *Streptococcus agalactiae* (group B streptococcus [GBS]) causes streptococcosis in fish (1), resulting in significant economic losses. For example, GBS was responsible for a massive disease outbreak in the Kuwait Bay in 2011, killing 2,500 metric tons of wild mullet (*Liza klunzingeri*) (2). In addition, large-scale streptococcal outbreaks occurred frequently in tilapia farms in China from 2009 to 2011, involving >95% of farms, with a cumulative mortality rate of 30 to 80% (3–5). GBS also causes meningitis (6–8), neonatal sepsis (9), and pneumonia (10) in humans. GBS is a major cause of bovine mastitis, a dominant health disorder affecting milk production (11, 12). A virulent GBS strain, *S. agalactiae* 138P, was isolated from infected Nile tilapia during a 2007 disease outbreak in Idaho. To develop an attenuated bacterial vaccine, a sparfloxacin resistance strategy was used to modify GBS strain 138P, resulting in the isolation of an attenuated GBS 138spar (13) that was 2,048-fold resistant to sparfloxacin. However, whether changes occurred at the genomic DNA level is unknown. Therefore, the whole-genome sequence of GBS 138spar was determined in this study.

The genome of GBS 138spar was sequenced using the Illumina 1500 HiSeq platform. BioNumerics (Applied Maths) was used to assemble a total of 2,743,316 sequence reads, with an average length of 100 bp. The whole genome of the virulent GBS strain 138P was reported recently (14). Using similar assembly methods as described previously (14), the whole genome of GBS 138spar was obtained, which is 1,838,126 bp in length. The RAST server (15) predicted 1,892 coding sequences, including all the subsystems reported in its parent strain, GBS 138P (14). RNAmmer (16) predicted 5 copies of 5S rRNA, 6 copies of 16S rRNA, and 6 copies of 23S rRNA, similar to those in the genome of GBS 138P and in the reference genome GBS 2-22 (GenBank accession no. FO393392). Compared to the whole genome of GBS 138P (14), GBS 138spar has a deletion >6 bp in 22 places. In addition, single-nucleotide changes (deletion, insertion, and point mutation) were found in 26 places in the GBS 138P genome compared to in the GBS 138spar genome.

### Nucleotide sequence accession number.

The complete genome sequence of *S. agalactiae* 138spar was deposited in GenBank under the accession no. CP007565.

## References

[1] Liu G Zhang W Lu C 2012 Complete genome sequence of *Streptococcus agalactiae* GD201008-001, isolated in China from tilapia with meningoencephalitis. J. Bacteriol. 194:6653. 10.1128/JB.01788-12 23144401 PMC3497532

[2] Glibert PM Landsberg JH Evans JJ Al-Sarawi MA Faraj M Al-Jarallah MA Haywood A Ibrahem S Klesius P Powell C Shoemaker C 2002 A fish kill of massive proportion in Kuwait Bay, Arabian Gulf, 2001: the roles of bacterial disease, harmful algae, and eutrophication. Harmful Algae 1:215–231. 10.1016/S1568-9883(02)00013-6

[3] Chen M Wang R Li LP Liang WW Li J Huang Y Lei AY Huang WY Gan X 2012 Screening vaccine candidate strains against *Streptococcus agalactiae* of tilapia based on PFGE genotype. Vaccine 30:6088–6092. 10.1016/j.vaccine.2012.07.044 22867719

[4] Chen M Li LP Wang R Liang WW Huang Y Li J Lei AY Huang WY Gan X 2012 PCR detection and PFGE genotype analyses of streptococcal clinical isolates from tilapia in China. Vet. Microbiol. 159:526–530. 10.1016/j.vetmic.2012.04.035 22677479

[5] Ye X Li J Lu M Deng G Jiang X Tian Y Quan Y Jian Q 2011 Identification and molecular typing of *Streptococcus agalactiae* isolated from pond-cultured tilapia in China. Fish. Sci. 77:623–632. 10.1007/s12562-011-0365-4

[6] Sanz-Rojas P Cabeza-Osorio L Hermosa C Serrano-Heranz R 2013 Acute meningitis by *Streptococcus agalactiae* in an immunocompetent male. Rev. Esp. Quimioter. 26:78–79 (In Spanish.) 23546471

[7] Reguera A González MÁ Sánchez A Aguayo C 2011 *Streptococcus agalactiae* meningitis in an immunocompetent male. Enferm. Infecc. Microbiol. Clin. 29:707–708 (In Spanish.) 10.1016/j.eimc.2011.05.020 21940070

[8] Martín Diaz RM Ruiz-Giardín JM Gonzalo Pascua S Canora Lebrato J 2010 Meningitis by *S.* *agalactiae* in a non-pregnant immunocompetent woman. Rev. Clín. Esp. 210:591–592 (In Spanish.) 10.1016/j.rce.2010.05.013 21036349

[9] Jawa G Hussain Z da Silva O 2013 Recurrent late-onset group B streptococcus sepsis in a preterm infant acquired by expressed breast milk transmission: a case report. Breastfeed Med. 8:134–136. 10.1089/bfm.2012.0016 23373437

[10] Villena Ruiz MA Olalla Sierra J de la Torre Lima J García-Alegría J 2009 *Streptococcus agalactiae* induced cavitated pneumonia. Rev. Clín. Esp. 209:252–254 (In Spanish.) 10.1016/S0014-2565(09)71245-7 19480785

[11] Katholm J Rattenborg E 2010 The surveillance program of *Streptococcus agalactiae* in Danish dairy herds, p 1989–2008 *In* Hillerton J, Proceedings of the 5th IDF Mastitis Conference. 2010. Mastitis Res. Into Practice. N Z Veterinary Association Vet. Learn. Christchurch, NZ.

[12] Richards VP Lang P Bitar PD Lefébure T Schukken YH Zadoks RN Stanhope MJ 2011 Comparative genomics and the role of lateral gene transfer in the evolution of bovine adapted *Streptococcus agalactiae*. Infect. Genet. Evol. 11:1263–1275. 10.1016/j.meegid.2011.04.019 21536150 PMC3139733

[13] Pridgeon JW Klesius PH 2013 Development of live attenuated *Streptococcus agalactiae* as potential vaccines by selecting for resistance to sparfloxacin. Vaccine 31:2705–2712. 10.1016/j.vaccine.2013.03.066 23583891

[14] Pridgeon JW Zhang D 2014 Complete genome sequence of a virulent *Streptococcus agalactiae* strain, 138P, isolated from diseased Nile tilapia. Genome Announc. 2(2):e00295-14. 10.1128/genomeA.00295-14 24744333 PMC3990749

[15] Aziz RK Bartels D Best AA DeJongh M Disz T Edwards RA Formsma K Gerdes S Glass EM Kubal M Meyer F Olsen GJ Olson R Osterman AL Overbeek RA McNeil LK Paarmann D Paczian T Parrello B Pusch GD Reich C Stevens R Vassieva O Vonstein V Wilke A Zagnitko O 2008 The RAST server: Rapid Annotations using Subsystems Technology. BMC Genomics 9:75. 10.1186/1471-2164-9-75 18261238 PMC2265698

[16] Lagesen K Hallin P Rødland EA Staerfeldt HH Rognes T Ussery DW 2007 RNAmmer: consistent and rapid annotation of ribosomal RNA genes. Nucleic Acids Res. 35:3100–3108. 10.1093/nar/gkm160 17452365 PMC1888812

